# A systematic review and meta-analysis on alcohol consumption and risk of endometriosis: an update from 2012

**DOI:** 10.1038/s41598-022-21173-9

**Published:** 2022-11-09

**Authors:** Letizia Li Piani, Francesca Chiaffarino, Sonia Cipriani, Paola Viganò, Edgardo Somigliana, Fabio Parazzini

**Affiliations:** 1 Department of Clinical Sciences and Community Health, Università degli Studi di Milano, Milan, Italy; 2 Infertility Unit, Fondazione IRCCS Ca’ Granda Ospedale Maggiore Policlinico, Via M. Fanti, 6, 20122 Milan, Italy; 3 Gynecology Unit, Fondazione IRCCS Ca’ Granda Ospedale Maggiore Policlinico, 20122 Milan, Italy

**Keywords:** Risk factors, Epidemiology, Infertility

## Abstract

Endometriosis is a complex and chronic disease, whose multifactorial nature has encouraged a deep investigation on the role of lifestyle factors. A strong association between alcohol intake and endometriosis risk has already been shown. We aimed to confirm this association, considering the updated literature. 23 eligible studies were identified through comprehensive literature search of PubMed and EMBASE (May 2012–October 2021). A borderline statistical significance was found comparing any alcohol consumption with no consumption (unadjusted OR 1.14; 95% CI: 0.99–1.31, p = 0.06), in contrast with a previous meta-analysis. However, we confirmed the significant association between moderate alcohol intake and endometriosis (unadjusted OR 1.22, 95% CI: 1.03–1.45, p = 0.02), also performing a sensitivity analysis (unadjusted OR 1.27, 95% CI: 1.04–1.54). Our partly divergent evidence reflects the tough challenge of isolating the impact of specific factors on the natural history of multifactorial diseases. Indeed, on one hand alcohol could be adopted by patients as a self-management therapy and on the other, it could favor the disease, promoting positive feedback with inflammatory mediators and oxidative stress. Our study encourages further investigation on the role of modifiable lifestyle factors and highlights the opportunity to adopt them to prevent or at least limit endometriosis progression.

## Introduction

Endometriosis is a progressive and hormone-related disease that strongly impacts on woman’s physical, mental, and social well-being^1^. Due to its debilitating nature and difficult clinical management, research has deeply investigated its possible pathogenesis. Despite decades of research efforts, the comprehension of endometriosis pathophysiology is still elusive and inconclusive^2–5^, but a unique cause seems not plausible. As for other chronic illnesses^6,7^, genetics and inflammation could be a common denominator of different pathogenetic mechanisms^8^. The pathway pain-stress-inflammation could play a key role in the development, progression, and exacerbations of endometriosis. Indeed, women with endometriosis seem to be caught in a “*vicious circle of high stress perception, inflammation and disease progression*”^9^. Besides, while intrauterine and neonatal exposure to prolonged physical stress stimuli could be linked to the future development^10^, in adults chronic stress might directly enhance the progression^11^. Inline, increased inflammatory markers have been documented not only in endometriosis lesions but also in peritoneal fluid and even in the peripheral blood of patients affected^12–14^.

A consequent important issue is to establish whether and how promoters of inflammation could influence endometriosis risk^15,16^. It is not surprising that modifiable lifestyle factors, such as diet, caffeine, environment, and smoking, all factors possibly associated with inflammation, have been explored in this regard^17–24^. Alcohol has already gained a certain attention. A meta-analysis from our group proved a significant correlation between alcohol intake and the occurrence of endometriosis^20^, based on the published papers until 2012^25–39^*.* However, given the preponderance of retrospective studies on the topic, at the time we claimed the need to confirm these findings. Therefore, the objective of this study was to verify the correlation between alcohol consumption and endometriosis risk, through a state-of-the-art systematic review and a meta-analysis.

## Methods

### Information sources

This systematic review was designed to meet the PRISMA (Preferred Reporting Items for Systematic Reviews and Meta-Analyses) guidelines^40^. Systematic research was conducted to search for relevant articles in which the impact of alcohol on endometriosis risk was discussed. The search terms “endometriosis” and “diet”, “nutrition”, “alcohol”, “vitamin”, “fat”, “vegetable,” were used as a combination of free text and as Medical Subject Heading (MeSH) terms (Pubmed) or Emtree terms (Embase) and temporally limited “from 2012/05/31 to 2021/10/11” (See search strategy in Supplementary file S1).

### Eligibility criteria

Inclusion criteria were:—case–control, cohort or cross-sectional study reporting original data from May 2012 to October 2021;—clinical or histological diagnosis of endometriosis;—presence of number or percentage of subjects with and without endometriosis according to alcohol intake;—full-length articles, published in English.

### Search strategy and data collection

**Figure 1:**
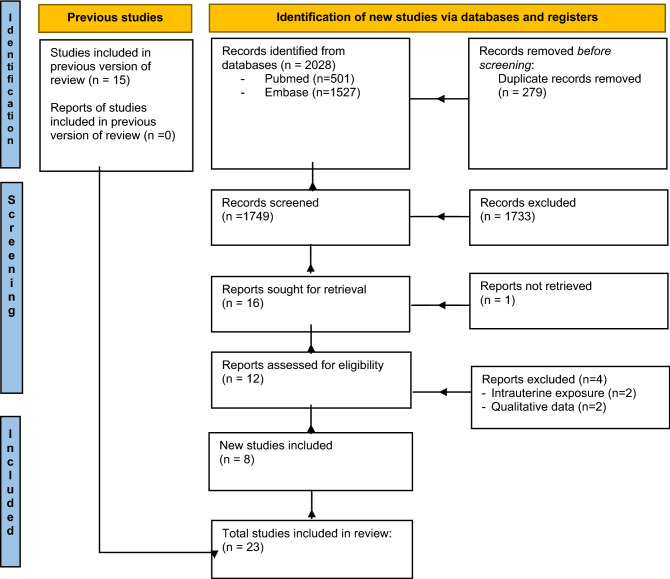
Flowchart of selection process according to PRISMA 2020 flow-diagram. It showed the study selection process. Only case–control, cohort and cross-sectional studies reporting original data were included. Conversely, case reports, case series and non-English language studies were excluded.

Data collection for our study followed the methodology of the previous one published in 2013^19^. Our research was registered in PROSPERO (ID: CRD42021282108). Figure 1 shows the selection procedure, according to PRISMA 2020^40^. First, two reviewers (LLP and FC) screened PUBMED and EMBASE to identify potential eligible studies. After excluding duplicated reports, they separately assessed all articles on title and abstract and selected relevant articles potentially meeting the inclusion criteria. They both read the full text of potentially eligible papers to assess whether they could be included. Full-text articles were reviewed, and discrepancies were discussed until consensus was reached among the authors. Exclusion reasons for potentially eligible studies were evaluation of intrauterine exposure to maternal alcohol intake or qualitative analysis of alcohol intake, in studies aimed to verify other associations.

Finally, data were extracted into Table 1, where we also considered the previous reports (n = 15). In the table, the following items were described: authors and publication date; country of origin; study design; number and age of participants; confounding factors; key findings.

**Table 1:** Main characteristics of considered studies.

Author, year	Country	Study design	Cases	Controls	Sample size cases/controls	Age (years)	Confounding factors considered in each study^a^	Key findings
Grodstein et al., 1994	USA, Canada	Hospital-based case–control	Women with primary infertility due to endometriosis	Fertile women	180/3833		Age, center, smoking habits, lifetime n. of sexual partner, contraception, BMI, exercise, coffee	Cases drank more alcohol than controls (40% vs 30%)
Signorello et al., 1997	USA	Hospital-based case–control	Women with infertility associated endometriosis	89 fertile women and 47 infertile women both without endometriosis	50/(89 and 47)	23–44	Age, education, height, weight, regularity of menstrual cycle, exercise smoking	Consumption of alcoholic beverages had a modest, non significant increase in endometriosis risk OR 2.0 (95%CI: 0.6–5.7)
Berubé et al., 1998	Canada	Case–control on prospective study	Infertile women for minimal or mild endometriosis (laparoscopically diagnosed)	Women with unexplained infertility	329/262	20–39		≥ 9 sd/month: OR 1.49 95%CI 0.93–2.37 No dose–response association
Pauwels et al., 2001	Belgium, Netherlands	Case–control	Infertile endometriosis women	Mechanical infertiles women	42/27	24–42	Age, BMI, ovulatory disfunction, smoking pattern, caffeine consumption	Similar number of alcohol consumers between cases and controls (3 vs 5)
Eskenazi et al., 2002	Italy	Cohort study	Women with endometriosis confirmed by surgery or ultrasound examination	Women without endometriosis confirmed by surgery or negative ultrasound examination	19/277	≤ 30 years old in 1976	Final models including only age because statistically significant	Lower alcohol intake in cases than in controls OR 0.39 (95%CI 0.11–1.38)
Hemmings et al., 2004	Canada	Hospital-based case–control	Women with endometriotic lesions at the time of surgery (surgery for diagnosis, fertility-regulating surgery, hysterectomy)	Women with no evidence of endometriotic lesion at surgery (surgery for diagnosis, fertility-regulating surgery, hysterectomy)	896/1881	Premenopausal age		No significant association between alcohol intake and endometriosis (≥ 7 sd/wk: OR 1.0; 95%CI 0.5–1.9)
Parazzini et al., 2004	Italy	Hospital-based case–control (from 2 studies)	Women with laparoscopically confirmed endometriosis	Women admitted for acute non-gynaecological, non-hormonal, non-neoplastic conditions	504/504	20–65	Age, calendar year, education, parity, BMI, study	No significant association between alcohol intake and endometriosis (“heavy intake”: OR 0.9; 95%CI 0.6–1.3)
Tsukino et al., 2005	Japan	Case–control	Women with stage II-IV endometriosis	Women without endometriosis or stage I endometriosis	58/81	20–45	Menstrual regularity, average cycle (days)	Alcohol intake in 39.6% of cases and in 49.4% of controls (p 0.45)
Buck Louis et al., 2007	USA	Hospital-based case–control in a cohort of women undergoing laparoscopy	Women with endometriosis in a cohort undergoing laparoscopy for any gynecologic indication including sterilization	Women without endometriosis from the same cohort of cases	32/52	18–40	In utero exposure, age, parity, smoking habit, caffeine intake	No significant association between alcohol intake and endometriosis risk (OR 0.4; 95%CI 0.07–1.8)
Heiler et al., 2007	Belgium	Matched Case–control	Women with peritoneal endometriosis (PE) or deep endometriotic nodules (DEN)	Women with no clinical suspicious of PE or DEN, without infertility, pelvic pain and dysmenorrhea and with normal pelvic examination, vaginal echography and serum CA-125 < 35U/ml	88 (PE), 88 (DEN)/88		None	Daily alcohol intake associated with deep endometriosis nodules (OR 4.58; 95%CI 1.80–11.62)
Matalliotakis et al., 2008	USA	Case–control in a retrospective review	Women with pelvic endometriosis who had undergone laparoscopy or laparotomy for pelvic pain or infertility within 6 years	Infertile women (tubal or male factor infertility)	535/200	15–56	None	Alcohol intake was more frequent in cases (44%) than in controls (28%) (p: 0.003)
Marino et al., 2009	USA	Case–control	Women with surgically confirmed endometriosis from the Group Health (GH) Cooperative	Women without endometriosis randomly selected from a list of GH during the same period	341/742	18–49	None	Cases were more likely than controls to be alcohol consumers (OR 1.45; 95%CI 1.07–1.97)
Nagle et al., 2009	Australia	Case–control	Women with surgically confirmed endometriosis	Women without endometriosis	268/244	18–55	None	Similar alcohol intake between cases and controls
Huang et al., 2010	Taiwan	Case–control	Women with endometriosis	Women without endometriosis	28/29	Mean age: cases = 34.3,controls = 36.2	Backward selection of confounders	Similar alcohol intake between cases and controls
Trabert et al., 2011	USA	Population-based case–control	Women with endometriosis (ICD9 = 617.0,.5,.8,.9) from the Group Health (GH) Cooperative	Women without endometriosis from the GH during the same period	284/660	18–49	None	Cases were more likely than controls to be alcohol consumers
**New contributions**								
Upson et al., 2013	USA	Population-based case–control study of endometriosis	Women with endometriosis confirmed by surgery	Women without endometriosis assessed by surgery	92/195	18–49		57% of cases reported current alcohol intake vs 42% controls
Prescott et al., 2016	USA	Prospective cohort study	Women with endometriosis confirmed by laparoscopy	Women without endometriosis assessed by surgery	658/22,581	24–44		Alcohol intake was 5.8 g/day and 6.0 g/day in cases and controls
Ricci et al., 2017	Italy	Hospital based case–control study of endometriosis	Infertile women with histologically confirmed endometriosis	Infertile women without endometriosis, admitted to hospital for acute conditions	90/90	17–76	Education, BMI, physical activity during adolescence	No significant increased endometriosis risk among alcohol users: (OR 1.48 95%CI: 0.68–2.79). No difference for the type of alcohol
Saha et al., 2017	Sweden	Population-based cross-sectional study	Women with endometriosis confirmed by medical records	Women without endometriosis	1228/27,594	20–65	Age, age at menarche, BMI, parity, OC use, infertility, coffee, smoking	No significant association between alcohol intake and endometriosis risk (< 4.5 sd/wk OR 0.9, 95%CI 0.76–1.07)
Ek et al., 2018	Sweden	Hospital-based case cohort study based on a study questionnaire	Women with endometriosis confirmed by surgery	Women from Malmo Diet and Cancer cardiovascular cohort	172/117	28–52	Age, education, occupation, marital status, smoking, physical activity, BMI	Alcohol intake was inversely associated with endometriosis (1-4sd: OR 0.16; 95%CI 0.09–0.30)
Hemmert et al., 2019	USA	Multicentric cohort study of women undergoing laparoscopy, regardless clinical indications	Women with endometriosis confirmed by surgery	Women without endometriosis diagnosis after surgery	190/283	18–44	Age, marital status, education, race/ethnicity, gravidity, BMI, relevant life-style factors, study site, pelvic pain	No association between endometriosis and alcohol consumption (OR 0.9, 95% CI 0.7, 1.3)
Schink et al., 2019	Germany	Retrospective case–control study	Women with endometriosis	Women without endometriosis	156/52	27/43	None	No significant higher alcohol intake in controls than in cases (9.8 ± 16.2 vs 6.3 ± 8.5 p 0.14)
Demézio da Silva et al., 2020	Brazil	Hospital-based case–control study of endometriosis	Women with endometriosis confirmed by surgery/MRI	Women with benign gynecological disease out of endometriosis, assessed by surgery	59/59	29–49	Age, BMI	Cases had lower alcohol intake than controls (25% vs 51%)

BMI, body mass index; CA, cancer antigen; DEN, deep endometriotic nodules; GH, group health; ICD-9, International Classification of Diseases, Ninth Revision; PE, peritoneal endometriosis; SD/WK: standard glass per week.

^a^The confounding factors column refers to the confounding factors considered in each paper. In our meta-analysis, our odd ratios were unadjusted for them.

### Statistical analysis

Statistical analyses were performed using Revman (Review Manager [Computer program], version 5.3; The Cochrane Collaboration, 2014) and STATA (STATA, version 10.0; StataCorp LP, College Station, TX, 2012). We pooled the unadjusted odds ratios (OR) by computing the random-effect model weighed for the inverse variance. To assess the heterogeneity across studies, we conducted a test based on the chi-square distribution. The funnel plot and Egger’s test were used to detect publication bias^41,42^. Two sensitivity analyses were also performed. In one, the data by Parazzini et al.^35^ were excluded because the reference category included women who consumed less than 0.5 drinks per week and not only the non-consumers. Furthermore, the category of moderate drinkers included women consuming relatively low amount (i.e. 0.5–8 drinks/week) in comparison to other studies. In another sensitivity analysis, the results by Bérubé^25^ were excluded because heavy drinkers were identified using a cut-off lower than the other studies (i.e., ≥ 9 drinks/month) and because they reported the prevalence OR (POR). We performed a further analysis excluding both studies (Bérubé et al. and Parazzini et al.) in order to evaluate the joint impact of these studies on the overall ORs.

### Quality assessment

The quality of the included studies was evaluated using the Newcastle–Ottawa Scale (NOS)^43^. Studies were evaluated according to three broad categories: selection of study groups, comparability of study groups, and assessment of outcome (cohort studies) or ascertainment of exposure (case–control or cross-sectional studies). The maximum score was 9.

## Results

### Systematic review

Selected articles are shown in Fig. 1. We identified 8 papers from May 2012 up to September 2021 to be assessed for the systematic review. Considering those selected for our 2013 study (n = 15)^20^, we counted a total number of 23 studies. In Table 1, we reported the main methodological characteristics of both the previous and the current selected articles, for a more complete information.

USA was the country for three papers^44–46^, one was conducted in Brazil^49^, and the other four were set in Europe^47,48,50,51^. The diagnosis of endometriosis was obtained by a surgical or clinical approach. Only Schink et al. ^50^ did not specify the diagnostic method to detect endometriosis. In Table 2, we detailed the cutoffs of alcohol drinking of the selected papers, according to the classification levels used in the previous meta-analysis^20^. Few articles reported the specific thresholds used^47,48,51^. In one study the category of “no alcohol intake” also included infrequent consumption (< 1 glass/week); for this reason, we excluded this study in the meta-analysis^48^. Moreover, in two papers, the cut-offs did not allow a precise classification between infrequent and moderate, while in another one between moderate and heavy consumption^48,51,52^.

**Table 2:** Classification of dose of alcohol drinking in different studies.

Author	Infrequent	Moderate/regular	Heavy
Grodstein et al., 1994		≤ 100 g/week = < 1 drink/day = < 30 drinks/month	> 100 g/week = ≥ 1 drink/day = ≥ 30 drinks/month
Signorello et al., 1997	< once/wk = < 4 drinks/month	≥ once/wk = ≥ 4 drinks/month	–
Bérubé et al., 1998	1–2 drinks/month	3–8 drinks/month	≥ 9 drinks/month
Pauwels et al., 2001	–	–	≥ 6 drinks/wk = ≥ 24 drinks/month
Hemmings et al., 2004	–	< 7 drinks/wk = < 30 drinks/month	≥ 7 drinks/wk = ≥ 30 drinks/month
Parazzini et al., 2004	–	0.5–8 drinks/wk §	≥ 8 drinks/week §
Tsukino et al., 2005		Weekly	Daily
Buck Louis et al., 2007	1–4 drinks/month	≥ 5 drinks/month	–
Heilier et al., 2007	< once/wk = < 4 drinks/month	Several times/wk = 4–29 drinks/month	Every day = ≥ 30 drinks/month
Nagle et al., 2009	€	€	
**New contributions**			
Ricci et al., 2017	–	≤ 7 drinks/wk	> 7 drinks/wk
Saha et al., 2017	–	≤ 4.5 drinks/wk	> 4.5 drinks/wk
Ek et al., 2018	–	≤ 4 drink/wk	> 4 drinks/wk
Hemmert et al., 2019	–	1–2 drinks/wk	≥ 3 drinks/wk

wk:week;

§ Tertile of intake. Reference category: < 0.5 drinks/week. A pure alcohol content was assumed in each type of drink (125 ml wine = 333 ml beer = 30 ml spirits).

€ Categories defined “Infrequent” and “Regular” by the Authors.

#### Cross-sectional studies

Only one of the newly selected articles had a cross-sectional design^51^. Saha et al., aimed to investigate the relationship between modifiable life-style factors and endometriosis in a cohort of 28,882 women: while a positive association between smoking or coffee intake with endometriosis was observed, they could not find a similar result considering alcohol consumption. Even taking into consideration the amount of alcohol per week, the association was not significant.

#### Cohort studies

Three cohort studies were identified after the previous review^20^. Prescott et al.^45^ designed a prospective cohort study to evaluate a possible link between endometriosis and infertility. Alcohol, expressed in terms of grams/day, was only mentioned as a covariate for their analysis. Hemmert et al.^46^ work stands out from the others for its nature: it was a multicenter prospective cohort design aimed to evaluate lifestyle exposure prior to endometriosis diagnosis. They observed null findings between endometriosis and alcohol intake, considering 473 women. In contrast, Ek et al.^48^ observed an inverse association between this habit and endometriosis, based on 172 women’s reported questionnaires.

#### Case–control studies

Most newly selected papers were case–control studies^44,47,49,50^. Both results from Schink et al.^50^ and Da Silva et al.^49^ agreed to deny an association between alcohol and endometriosis; indeed, they both observed that alcohol intake tended to be higher in unaffected patients. In contrast, Ricci et al. found an increased endometriosis risk among alcohol users^47^.

### Meta-analysis

**Figure 2:**
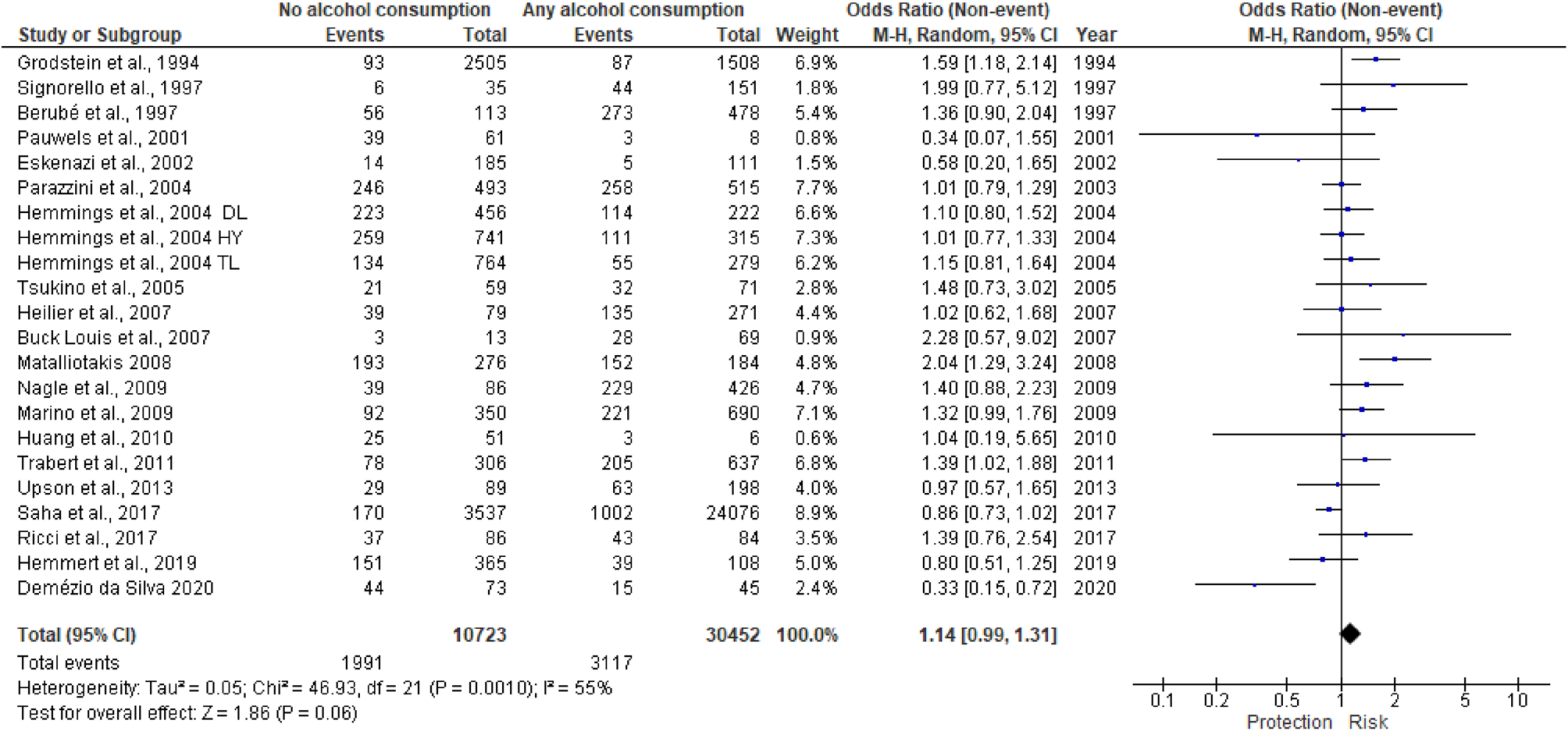
Any versus no alcohol consumption. It presented the summary results of the analyses of any intake vs no alcohol intake. In this figure, endometriosis risk due to any alcohol consumption is expressed in terms of unadjusted odds ratio (OR).

A total of 22 papers were included in the meta-analysis. Figure 2 depicted the study-specific and pooled ORs for any versus no alcohol intake. We were not able to find an overall statistically significant association between any alcohol consumption and endometriosis risk (unadjusted OR 1.14; 95% CI: 0.99–1.31) although a borderline statistical significance was observed (p = 0.06).

**Figure 3:**
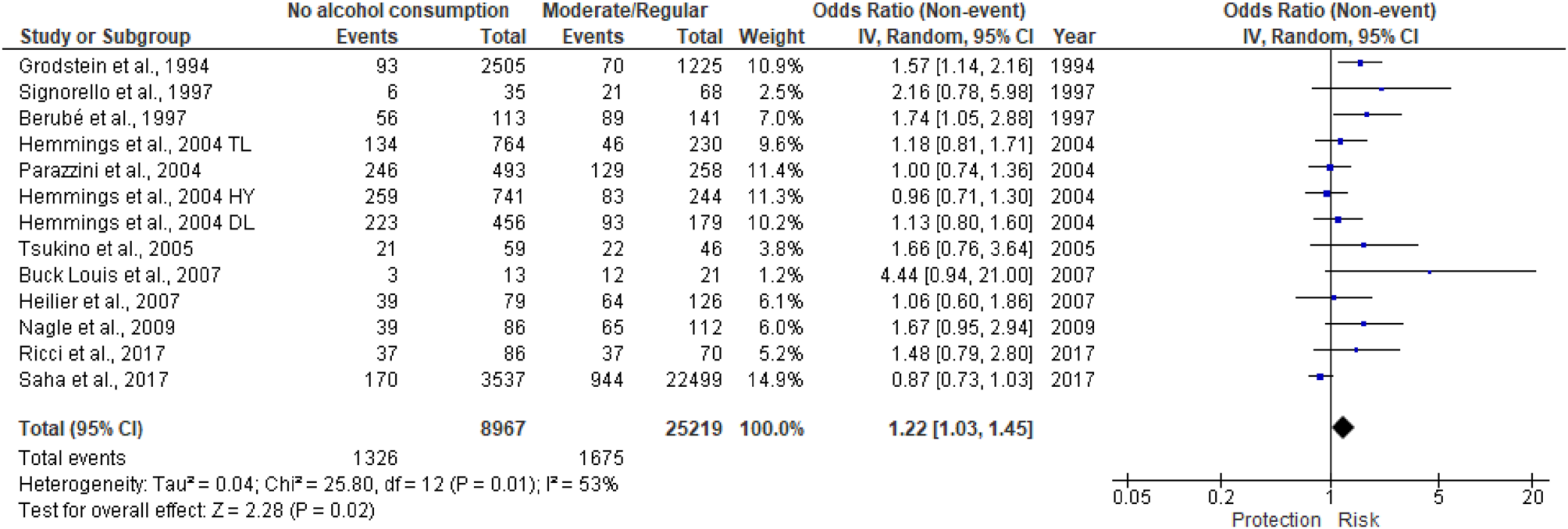
Moderate/regular versus no alcohol consumption. It presented the results of the analyses of moderate intake vs no alcohol intake. As in Fig. 2, endometriosis risk due to regular alcohol consumption is expressed in terms of unadjusted odds ratio (OR).

**Figure 4:**
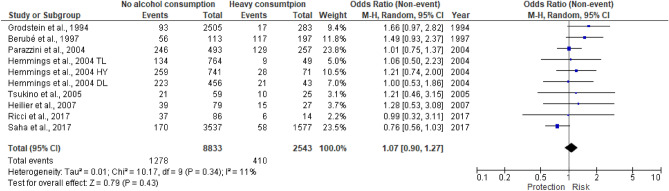
Heavy versus no alcohol consumption. It presented the results of the analyses of heavy intake vs no alcohol intake. As in Fig. 2, endometriosis risk due to heavy alcohol consumption is expressed in terms of unadjusted odds ratio (OR).

We also evaluated the effect of alcohol intake according to the number of drinks (Figs. 3, 4). None of the new papers reported a consumption attributable to the “infrequent” category, as adopted in the meta-analysis of Parazzini et al.^20^. For this reason, we did not report the forest plot. Thus, considering “infrequent” vs no alcohol intake, the OR remained 1.14 (95%CI 0.86–1.52)^20^. We found out a statistically significant association only when comparing moderate versus no alcohol consumers (p = 0.02) with a summary OR of 1.22 (95% CI, 1.03–1.45). In contrast, in case of heavy alcohol intake, the result was not significant, with an OR of 1.07 (95%CI, 0.90–1.27).

We performed two sensitivity analyses. In one, we excluded the data by Parazzini et al.^35^: the OR for moderate versus no alcohol consumption was 1.27 (95% CI, 1.04–1.54). In the second sensitivity analysis, the OR estimate for heavy versus no alcohol consumption was 1.01 (95% CI, 0.85–1.19) when we excluded the study of Bérubé et al.^25^. To assess the joint impact of the data by Bérubé et al. and Parazzini et al., we performed further analyses by excluding both the studies: the overall ORs were 1.14 (95% CI, 0.97–1.33), 1.22 (95% CI, 1.01–1.49) and 1.03 (95% CI, 0.83–1.28) for any, moderate and heavy versus no alcohol consumption, respectively.

Data from Schink et al.^50^ and Prescott et al.^45^ could not be included in the meta-analysis as they reported the value of alcohol intake as means of grams per day. Intriguingly, in both papers, unaffected women were more likely to drink alcohol than affected ones, even if without statistically significance*.* Data were then analyzed according to the time of alcohol intake (current, former or both). Compared to the previous meta-analysis, only one additional study provided information on this aspect^44^ (Table 3). However, compared to our previous analysis^20^, the added data did not significantly change the summary ORs. The updated estimates are reported in Table 3.

**Table 3:** Alcohol drinking and risk of endometriosis according to time of intake.

Author	Never^a^	Current	Former	Current/former
	Cases/controls	Cases/controls OR (95% IC) IC)	Cases/controls OR (95% IC)	Cases/controls OR (95% IC)
Eskenazi et al., 2002	14/171	3/95 0.39 (0.11–1.38)	2/11 2.22 (0.45–11.02)	–
Matalliotakis,2008	193/83	–	–	152/32 2.04 (1.29–3.24)
Marino, 2009	92/258	159/307 1.45 (1.07–1.97)	62/162 1.07 (0.74–1.57)	–
Huang, 2010	25/26	–	–	3/3
Trabert, 2011	78/228	154/292 1.54 (1.12–2.13)	51/140 1.06 (0.71–1.61)	–
**New contributions**				
Upson et al., 2013	29/60	52/90	11/45	
Pooled OR		1.39 (1.1–1.7)	1.16 (0.90–1.50)	1.95 (1.26–3.04)

OR, Odds ratio; IC, confidence interval.

^a^Reference category.

Finally, only Ricci et al.^47^ exploited the effect of different types of beverages, observing a positive, although not significant, association between alcohol intake and endometriosis risk, regardless of wine or beer or spirit.

Supplementary Figure S1 showed the funnel plot for any versus no alcohol consumption. There was no asymmetry in the funnel plot, thus suggesting the absence of publication bias; the Egger test was not significant.

Evaluation of the study quality according to the Newcastle–Ottawa Scale^43^, was reported in Supplementary Table S3. Using the NOS tool, high study quality (scale = 7–9) was detected in 16 out of 19 case–control studies and in all the cohort and cross-sectional studies.

## Discussion

Alcohol consumption in endometriosis has deserved particular attention for many reasons. As summed up by Parazzini et al., alcohol may interfere with estrogen production, that is critically linked to endometriosis^20,25,52,53^. Moreover, alcohol could be implied in the vicious circle “pain-stress-inflammation”^9,54^. In 2021, one of the early effects of COVID-19 pandemic was an increase of alcohol consumption for 29.2% endometriosis patients^55^. Notably, psychiatric disorders (bipolar, depressive, anxiety, and stress-related syndromes) and endometriosis may be intertwined^56–58^ so that endometriosis patients may more likely suffer of these conditions^59^. However, it is not clear whether psychiatric comorbidities burden on endometriosis or whether they are the consequence of painful symptoms. Nevertheless, it is not difficult to figure out that chronic pain as well as impaired psychological well-being may encourage alcohol misuse^60,61^. Alcohol consumption could be framed as a possible wrong self-medication to cope with either stressful or painful events. Indeed, Gao et al., observed a higher risk of developing alcohol/drug dependence disorders (HR 1.93; 95%CI, 1.71–2.18) rather than other psychiatric conditions in endometriosis population. On the other hand, they also observed the opposite: alcohol/drug dependence disorders were at higher risk for a subsequent endometriosis diagnosis (HR 1.94; 95%CI, 1.84–2.04)^56^.

To note, alcohol metabolism influences pro-inflammatory pathways and oxidative stress^62–64^. Collecting all these premises, alcohol could be involved in endometriosis in two different ways: on one hand, it could be an effect of the disease, adopted by patients as a self-management therapy for pain and stressful events or as an expression of psychiatric comorbidity; on the other one, alcohol habit could favor the disease, promoting the positive feedback with inflammatory mediators and oxidative stress.

As already mentioned, our group previously found a significant positive association between alcohol consumption and endometriosis risk^20^. Our goal was to corroborate this result, updating data with the recent literature.

Excluding the two papers where alcohol was expressed in grams^44,45^, among the newly selected papers we could observe contrasting results. While Ricci et al. ^47^ reported an increased, although not significant, endometriosis risk according to alcohol intake (OR 1.48, 95%CI 0.68–2.79), Ek et al.^48^, Schink aet al.^49^ and da Silva et al.^50^ agreed that unaffected patients tended to be more likely alcohol users. Hemmert et al.^46^ and Saha et al.^51^ found no association between this habit and endometriosis risk.

Overall, in our meta-analysis considering aggregated data to date, although an increased risk of endometriosis was confirmed among any alcohol users, the finding was only of borderline statistical significance and the OR estimate was lower than in our previous analysis.

To explain these not totally consistent findings, some differences with the previous results should be considered. In the present meta-analysis, a higher number of prospective studies have been included. Only one of the selected recent papers was a cross-sectional study^51^. Thus, the possible bias derived by ascertaining exposure and outcome at the same time was reduced. In the previous review, the higher proportion of retrospective case–control studies could favor the introduction of selection and recall bias. Collecting lifestyle information before outcome assessment could limit recall bias. Hemmert et al.^46^ used this approach, denying any association between alcohol and endometriosis occurrence. Indeed, going deeper into the concept of exposure, Wolff et al. assessed in utero exposures and the risk of endometriosis diagnosis. Intriguingly, though not significantly, affected women were less likely to have been exposed to alcohol during pregnancy^10^.

Interestingly, we confirmed a significant relation between a moderate/regular alcohol intake and endometriosis, with a significant OR of 1.22 (95% CI: 1.03–1.45, p = 0.02). This finding could be in line with the possible double role of alcohol in the natural history of the disease, as described before. Furthermore, it is reasonable to infer that this result is still related to results from the studies included in the previous meta-analysis^20^. Indeed, we have added only 3 papers to the other 11 previous works reporting data for the subgroup of “moderate intake”. In other words, findings reported before 2012 could have been robust enough to provide still significant results for the subgroup of “moderate intake”, while they could have been diluted in the overall group of “any intake” by the addition of the more recent findings.

We recognize that our study has some limitations that must be addressed. In some papers, endometriosis diagnosis was not confirmed by surgery. This choice may have allowed the inclusion of affected women in the control group; however, the general prevalence of the disease is less than 5%^2^ and this ascertainment bias cannot be expected to have mainly distorted the results. Moreover, self-reported alcohol intake may have introduced a further bias, especially in the evaluation of number of usual drinks. On the other hand, the funnel plot and the Egger test for funnel plot asymmetry did not show evidence of publication bias.

The difficulty in establishing whether alcohol exposure precedes endometriosis represents the biggest limitation for drawing definitive conclusions and still constitutes the real challenge. This issue was similarly reported in several works, concerning other modifiable factors. In two independent reviews, both Parazzini et al.^19^ and later Osmanlioglu et al.^22^ agreed that evidence supporting a significant association between diet and endometriosis is equivocal. Polak et al.^21^ could not take out any significant conclusions about the relation between environment and endometriosis risk. On the other hand, higher concentrations of trans-nonachlor, and dioxin-like toxic equivalents, together with an increased inflammatory profile have been associated with higher risk of endometrioma^65^. Environmental exposure remains a major and unsolved issue^66^.

In line with these papers, our work reflects the tough challenge of isolating the role of specific factors in the natural history of multifactorial diseases, such as endometriosis. Nevertheless, never as now the investigation of modifiable lifestyle factors is urgent for a new integrated therapeutic approach.

From our meta-analysis, we could confirm a significant association between moderate alcohol intake and endometriosis but the strength of our previous results could not be proved considering the other categories. Despite this could be due to some methodological differences (i.e. the nature of published studies), establishing the role of alcohol in the pathogenesis and progression of disease remains an undisputed need.

## Supplementary Information

Supplementary Figure S1.

Supplementary Information 2.

Supplementary Table S1.

## Data Availability

All data generated or analyzed during this study are included in this published article and in its supplementary information file.
