# Supplementary material for: A systematic review and meta-analysis on alcohol consumption and risk of endometriosis: an update from 2012
Source: Sci Rep. 2022 Nov 9;12:19122. doi: 10.1038/s41598-022-21173-9 (PMC9645754; doi:10.1038/s41598-022-21173-9)

A systematic review and meta-analysis on alcohol consumption and risk of endometriosis: an update from 2012

Letizia LI PIANI, Francesca CHIAFFARINO,Sonia CIPRIANI, Paola VIGANO', Edgardo SOMIGLIANA, Fabio PARAZZINI

SUPPLEMENTARY FIGURE S1. Funnel plot of any vs no alcohol consumption

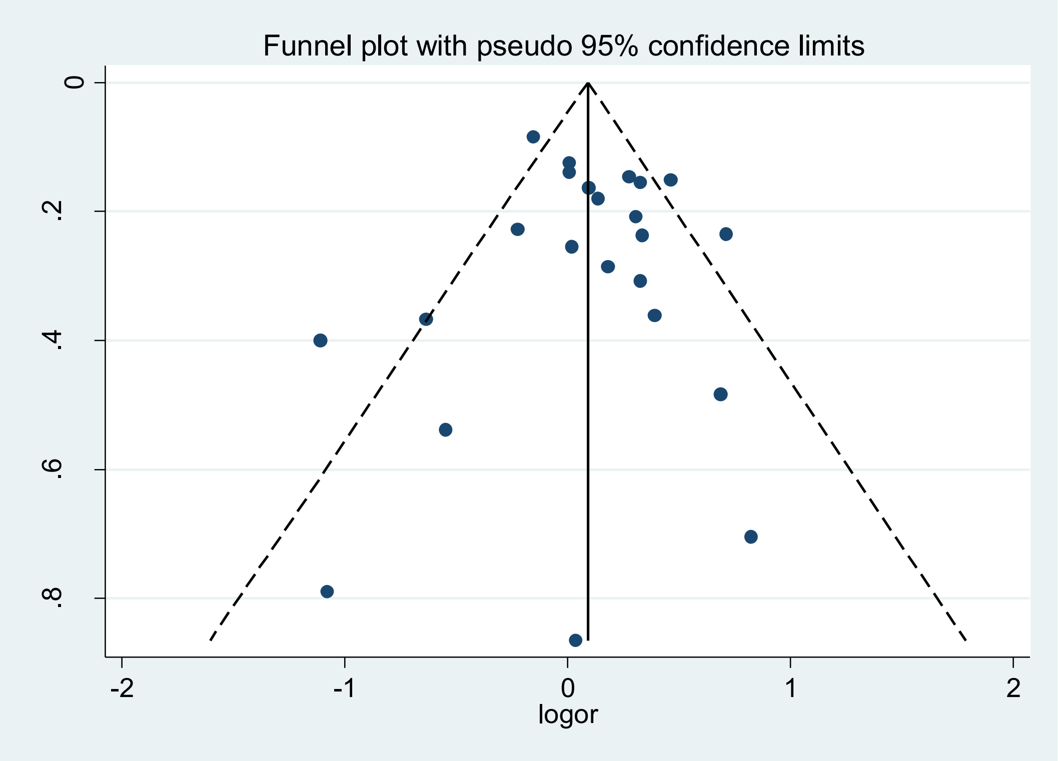

Supplement: Supplementary file 1 — Supplementary Figure S1. [file 41598_2022_21173_MOESM1_ESM.pdf]
