# Supplementary material for: A systematic review and meta-analysis on alcohol consumption and risk of endometriosis: an update from 2012
Source: Sci Rep. 2022 Nov 9;12:19122. doi: 10.1038/s41598-022-21173-9 (PMC9645754; doi:10.1038/s41598-022-21173-9)
Supplement: Supplementary file 2 — Supplementary Information 2. [file 41598_2022_21173_MOESM2_ESM.pdf]

A systematic review and meta-analysis on alcohol consumption and risk of endometriosis: an update from 2012

Letizia LI PIANI, Francesca CHIAFFARINO,Sonia CIPRIANI, Paola VIGANO’, Edgardo SOMIGLIANA, Fabio PARAZZINI

Supplementary file S1. Search strategy

PUBMED

("diet"[MeSH Terms] OR "diet"[All Fields] OR ("nutrition s"[All Fields] OR "nutritional status"[MeSH Terms] OR ("nutritional"[All Fields] AND "status"[All Fields]) OR "nutritional status"[All Fields] OR "nutrition"[All Fields] OR "nutritional sciences"[MeSH Terms] OR ("nutritional"[All Fields] AND "sciences"[All Fields]) OR "nutritional sciences"[All Fields] OR "nutritional"[All Fields] OR "nutritionals"[All Fields] OR "nutritions"[All Fields] OR "nutritive"[All Fields]) OR ("alcohol s"[All Fields] OR "alcoholate"[All Fields] OR "alcoholates"[All Fields] OR "alcoholic s"[All Fields] OR "alcoholics"[MeSH Terms] OR "alcoholics"[All Fields] OR "alcoholic"[All Fields] OR "alcoholism"[MeSH Terms] OR "alcoholism"[All Fields] OR "alcoholisms"[All Fields] OR "alcoholism s"[All Fields] OR "alcoholization"[All Fields] OR "alcohols"[MeSH Terms] OR "alcohols"[All Fields] OR "ethanol"[MeSH Terms] OR "ethanol"[All Fields] OR "alcohol"[All Fields]) OR ("vitamin s"[All Fields] OR "vitamine"[All Fields] OR "vitamines"[All Fields] OR "vitamins"[Pharmacological Action] OR "vitamins"[MeSH Terms] OR "vitamins"[All Fields] OR "vitamin"[All Fields]) OR "fat"[All Fields] OR ("vegetables"[MeSH Terms] OR "vegetables"[All Fields] OR "vegetable"[All Fields])) AND ("endometriosis"[MeSH Terms] OR "endometriosis"[All Fields] OR "endometrioses"[All Fields]) AND 2012/05/31:2021/10/11[Date - Create]

EMBASE

('diet'/de OR 'diet' OR 'nutrition'/de OR 'nutrition' OR 'alcohol'/de OR 'alcohol' OR 'drinking behaviour'/de OR 'fat'/de OR 'fat' OR 'vegetable'/de OR 'vegetable' OR 'vitamin'/de OR 'vitamin' OR 'diet'/exp OR 'nutrition'/exp OR 'alcohol'/exp OR 'fat'/exp OR 'vegetable'/exp OR 'vitamin'/exp) AND ('endometriosis'/de OR 'endometriosis' OR 'endometriosis'/exp) AND [31-05-2012]/sd NOT [12-10-2021]/sd
