# Supplementary material for: A systematic review and meta-analysis on alcohol consumption and risk of endometriosis: an update from 2012
Source: Sci Rep. 2022 Nov 9;12:19122. doi: 10.1038/s41598-022-21173-9 (PMC9645754; doi:10.1038/s41598-022-21173-9)
Supplement: Supplementary file 3 — Supplementary Table S1. [file 41598_2022_21173_MOESM3_ESM.pdf]

**A systematic review and meta-analysis on alcohol consumption and risk of endometriosis: an update from 2012**

Letizia LI PIANI, Francesca CHIAFFARINO,Sonia CIPRIANI, Paola VIGANO', Edgardo SOMIGLIANA, Fabio PARAZZINI

**Supplementary TABLE S1.** Newcastle Ottawa scale

| CASE-CONTROL STUDIES | Question # | Selection | Question # | Comparability | Question # | Exposure | Study quality | (Refs.) |
|---|---|---|---|---|---|---|---|---|
| Grodstein et al., 1994 | 1<br>2<br>3<br>4 | *<br>*<br>-<br>* | 1<br>2 | *<br>* | 1<br>2<br>3 | *<br>*<br>* | 8/9 | (28) |
| Signorello et al., 1997 | 1<br>2<br>3<br>4 | *<br>*<br>-<br>* | 1<br>2 | *<br>* | 1<br>2<br>3 | *<br>*<br>* | 8/9 | (37) |
| Berubé et al., 1998<br>POR | 1<br>2<br>3<br>4 | *<br>*<br>-<br>* | 1<br>2 | *<br>* | 1<br>2<br>3 | *<br>*<br>* | 8/9 | (25) |
| Pauwels et al., 2001 | 1<br>2<br>3<br>4 | *<br>*<br>-<br>* | 1<br>2 | *<br>* | 1<br>2<br>3 | -<br>*<br>* | 7/9 | (36) |
| Hemmings et al., 2004 | 1<br>2<br>3<br>4 | *<br>*<br>-<br>* | 1<br>2 | *<br>* | 1<br>2<br>3 | *<br>*<br>* | 8/9 | (30) |
| Parazzini et al., 2004 | 1<br>2<br>3<br>4 | *<br>*<br>-<br>* | 1<br>2 | *<br>* | 1<br>2<br>3 | *<br>*<br>* | 8/9 | (35) |
| Tsukino et al., 2005 | 1<br>2<br>3 | *<br>*<br>- | 1<br>2 | *<br>* | 1<br>2<br>3 | *<br>*<br>* | 7/9 | (39) |

| | | | | | | | | |
|---|---|---|---|---|---|---|---|---|
| | 4 | - | | | | | | |
| Buck Louis et al., 2007 | 1<br>2<br>3<br>4 | *<br>*<br>-<br>* | 1<br>2 | *<br>* | 1<br>2<br>3 | *<br>*<br>* | 8/9 | (26) |
| Heiler et al., 2007 | 1<br>2<br>3<br>4 | *<br>*<br>*<br>* | 1<br>2 | -<br>- | 1<br>2<br>3 | *<br>*<br>* | 7/9 | (29) |
| Matalliotakis et al., 2008 | 1<br>2<br>3<br>4 | *<br>*<br>-<br>* | 1<br>2 | -<br>- | 1<br>2<br>3 | *<br>*<br>* | 6/9 | (33) |
| Marino et al., 2009 | 1<br>2<br>3<br>4 | *<br>*<br>*<br>* | 1<br>2 | -<br>- | 1<br>2<br>3 | *<br>*<br>* | 7/9 | (32) |
| Nagle et al., 2009 | 1<br>2<br>3<br>4 | *<br>*<br>*<br>* | 1<br>2 | -<br>- | 1<br>2<br>3 | *<br>*<br>* | 7/9 | (34) |
| Huang et al., 2010 | 1<br>2<br>3<br>4 | *<br>*<br>-<br>* | 1<br>2 | *<br>* | 1<br>2<br>3 | *<br>*<br>* | 8/9 | (31) |
| Trabert et al., 2011 | 1<br>2<br>3<br>4 | *<br>*<br>*<br>* | 1<br>2 | -<br>- | 1<br>2<br>3 | *<br>*<br>* | 7/9 | (38) |
| Upson et al., 2013 | 1<br>2<br>3<br>4 | *<br>*<br>*<br>* | 1<br>2 | *<br>* | 1<br>2<br>3 | *<br>*<br>* | 9/9 | (44) |
| Ricci et al., 2017 | 1<br>2<br>3 | *<br>*<br>- | 1<br>2 | *<br>* | 1<br>2<br>3 | *<br>- | 6/9 | (47) |

| | | | | | | | | |
|---|---|---|---|---|---|---|---|---|
| | 4 | * | | | | | | |
| Ek et al., 2018 | 1<br>2<br>3<br>4 | *<br>*<br>*<br>* | 1<br>2 | *<br>* | 1<br>2<br>3 | *<br>*<br>* | 9/9 | (48) |
| Schink et al., 2019 | 1<br>2<br>3<br>4 | *<br>*<br>-<br>* | 1<br>2 | -<br>- | 1<br>2<br>3 | *<br>*<br>* | 6/9 | (50) |
| Demézio da Silva et al., 2020 | 1<br>2<br>3<br>4 | *<br>*<br>-<br>* | 1<br>2 | *<br>* | 1<br>2<br>3 | *<br>*<br>* | 8/9 | (49) |
| **COHORT STUDIES** | **Question #** | **Selection** | **Question #** | **Comparability** | **Question #** | **Outcome** | **Study quality** | **(Refs.)** |
| Eskenazi et al., 2002 | 1<br>2<br>3<br>4 | -<br>*<br>*<br>* | 1<br>2 | *<br>* | 1<br>2<br>3 | *<br>*<br>* | 8/9 | (27) |
| Prescott et al., 2016 | 1<br>2<br>3<br>4 | -<br>*<br>*<br>* | 1<br>2 | *<br>* | 1<br>2<br>3 | *<br>*<br>* | 8/9 | (45) |
| Hemmert et al., 2019 | 1<br>2<br>3<br>4 | *<br>*<br>*<br>* | 1<br>2 | *<br>* | 1<br>2<br>3 | *<br>*<br>* | 9/9 | (46) |
| **CROSS-SECTIONAL STUDIES** | **Question #** | **Selection** | **Question #** | **Comparability** | **Question #** | **Exposure** | **Study quality** | **(Refs.)** |
| Saha et al, 2017 | 1<br>2<br>3<br>4 | *<br>*<br>*<br>* | 1<br>2 | *<br>* | 1<br>2<br>3 | *<br>** | 9/9 | (51) |
